# Targeting the Vasculature of Colorectal Carcinoma with a Fused Protein of (RGD)_3_-tTF

**DOI:** 10.1155/2013/637086

**Published:** 2013-06-18

**Authors:** Zheng-jie Huang, Yilin Zhao, Wei-yuan Luo, Jun You, Shui-wen Li, Wen-cheng Yi, Sheng-yu Wang, Jiang-hua Yan, Qi Luo

**Affiliations:** ^1^Department of Surgical Oncology, First Affiliated Hospital of Xiamen University, Xiamen, Fujian Province 361003, China; ^2^Department of Hepatobiliary Surgery, Zhongshan Hospital of Xiamen University, Xiamen, Fujian Province 361004, China; ^3^Cancer Research Center of Medical School, Xiamen University, Xiamen, Fujian Province 361102, China

## Abstract

*Purpose*. Truncated tissue factor (tTF) fusion protein targeting tumor vasculature can induce tumor vascular thrombosis and necrosis. Here, we generated (RGD)_3_-tTF in which three arginine-glycine-aspartic (RGD) targeting integrin *α*_*v*_ *β*_3_ and tTF induce blood coagulation in tumor vessels. *Methods*. The bioactivities of (RGD)_3_-tTF including coagulation activity, FX activation, and binding with integrin *α*_*v*_ *β*_3_ were performed. The fluorescent labeled (RGD)_3_-tTF was intravenously injected into tumor-bearing mice and traced in vivo. The tumor growth, volume, blood vessel thrombosis, tumor necrosis, and survival time of mice treated with (RGD)_3_-tTF were evaluated. *Results*. The clotting time and FX activation of (RGD)_3_-tTF were similar to that of TF (*P* > 0.05) but different with that of RGD (*P* < 0.05). (RGD)_3_-tTF presented a higher binding with *α*_*v*_ *β*_3_ than that of RGD and TF at the concentration of 0.2 *μ*mol/L (*P* < 0.05). (RGD)_3_-tTF could specifically assemble in tumor and be effective in reducing tumor growth by selectively inducing tumor blood vessels thrombosis and tumor necrosis which were absent in mice treated with RGD or TF. The survival time of mice treated with (RGD)_3_-tTF was higher than that of mice treated with TF or RGD (*P* < 0.05). *Conclusion*. (RGD)_3_-tTF may be a promising strategy for the treatment of colorectal cancer.

## 1. Introduction

Traditionally, treatment approaches for cancer therapy have directly focused on destroying cancer cells and tissue. Recently, another treatment approach has achieved great attention. Rather than directly targeting the neoplastic cells, this strategy tried to block the tumor's blood support system by targeting the tumor vasculature [1, 2]. 

It is known that most tumors remain being quiet and fail to grow over a few millimeters in size without angiogenesis [3]. Blood supply is a key factor in cancer tissue surviving, progressing, and spreading. The therapeutic potential of targeting the tumor vasculature is quite clear and promising [2]. Drugs which are capable of specifically targeting tumor blood vessels have been being developed and explored. Many of these drugs have been for clinical evaluation. The strategies directly targeting the blood vessel can not only be used alone but also be used in combination with conventional anticancer treatments [4, 5]. 

Tissue factor (TF) is a transmembrane glycoprotein and is the originating factor of blood coagulation cascade [6]. The truncated tissue factor (tTF) is the extracellular domain of tissue factor and is less 100000-fold than tissue factor in activating blood coagulation [7]. The complex of tTF and blood coagulation factor VII (F VII) cannot effectively activate coagulation factor X to trigger the blood coagulation because its incomplete structure is unable to bind with cell membrane [8, 9]. The fusion protein consists of the extracellular domain of tissue factor (truncated tissue factor (tTF)) and the antibody which can selectively bind to tumor vasculature [9]. The molecules which can specifically bind with the markers on endothelium of tumor blood vessels could be used as the carriers of tTF for improving their binding with endothelium cells and enhancing their coagulation capacity [10]. The function of tTF in vector tTF to activate coagulation FX will restore and is capable of inducing tumor vascular thrombosis and leading to tumor necrosis [11, 12]. Studies indicated that there were some disadvantages of using antibodies of tumor vascular markers as the specific vector of tTf [8]. The disadvantages are mainly reflected in the following two aspects. Firstly, as the antibody molecule is relatively large, it easily exerts steric hindrance which will affect the binding capacity of tTF with factor VII and factor X, and thus the efficiency of inducing tumor vascular thrombosis is reduced [13]. Secondly, antibody-tTF fusion protein can theoretically be taken in by the liver, spleen, and other reticuloendothelial systems, so there is potential risk of causing thrombosis in these organs [8]. 

Endothelium of blood vessels in colorectal cancer tissues presents an important target for colorectal cancer therapy [14]. Vascular targeting requires the identification of target molecules that are present on vascular endothelium at sufficient density in solid tumors but are absent from endothelial cells in normal tissues [15]. Such molecules could be used to target the vascular endothelium of the tumor rather than the tumor cells themselves. Promising candidate molecules include anti-vascular endothelial cell adhesion molecule 1 (VCAM-1) antibody and anti-vascular endothelial growth factor (VEGF) antibody [16, 17]. As integrin *α*_*v*_ *β*_3_ is highly expressed by vascular endothelial cells in colorectal cancer tissues, it could be served as a tumor vascular target for molecular therapy of colorectal cancer [18, 19]. 

Studies indicated that repeated RGD sequences had a higher affinity on integrin *α*_*v*_ *β*_3_ receptors than the single RGD sequence had [20]. Thus, in this study, we produced the fusion protein (RGD)_3_-tTF which was consisted of tTF and triple peptides of RGD as the carrier of tTF for targeting tumor vasculature in the treatment of mice colorectal carcinoma. 

## 2. Materials and Methods 

### 2.1. Primers Preparation

All primers were synthesized by Sangon Biotech (Shanghai) Co. Ltd. Primers for tTF cDNA were 5′-TCTGGCACTACAAATACTGTGGC-3′ (P_1_, upstream primer) and 5′-TTCTCTGAATTCCCCTTTCTCC-3′ (P_2_, downstream primer). P_3_ was designed according to the literature [8]. P_3_ was overlapping oligonucleotides which was 5′-CATACCATGGGC(TGCGATTGTCGCGGAGATTGCTTCTGCGGTGGAGGCGGGTCT)_3_ **TCTGGCAC TACAAATAC**-3′ (the straight line was RGD-4C sequence, and bold was 5′ end sequence of tTFgene). Primers containing endonuclease sites of Nco I and Xho I were 5′-CATACCATGGGCTGCGATTGTC-3′ (P_4_ upstream primer) and 5′-CTACCTCGAGTTCTCTGAATTCCCCTTTCTCC-3′ (P_5_ downstream primer).

### 2.2. Construction of Fusion Gene of (RGD)_3_-tTF

The gene of (RGD)_3_-tTF was amplified by PCR. Briefly, tTF-pSK(+) was used as the template, P_1_ and P_2_ were used as primers, and the tTF gene was amplified by using routine PCR. Then, the amplified tTF gene and P_3_ were added to PCR reaction system and annealed to achieve the fusion gene template of (RGD)_3_-tTF. P_4_ and P_5_ were then added to the PCR reaction system to produce the fusion gene of (RGD)_3_-tTF containing Nco I and Xho I endonuclease sites in the 5′ and 3′ ends, respectively. 

### 2.3. Preparation of Vector Containing (RGD)_3_-tTF Gene

By using the DNA Ligation Kit (NEB), the cDNA of (RGD)_3_-tTF was cloned into the expression vector pET22b(+) (Novagen) containing Nco I and Xho I endonuclease sites. Briefly, the fusion genes of (RGD)_3_-tTF and pET22b(+) were digested with the Nco I and Xho I restriction enzyme. The (RGD)_3_-tTF gene was then cloned into pET22b(+) to produce an expression vector encoding a fusion protein. The (RGD)_3_-tTF-pET22b(+) vector was then transferred into *E. coli* (*E. coli*) BL21 (DE_3_) and cultured in ampicillin plate for selective screening. The positive clones were used for (RGD)_3_-tTF sequencing analysis. 

### 2.4. The Expression and Purification of Fusion Protein

To amplify the *E. coli* colonies containing the reconstructed vetor of (RGD)_3_-tTF-pET22b(+), the (RGD)_3_-tTF fusion protein was expressed in *Escherichia coli* strain and purified by nickel affinity chromatography column purification according to the manufacturer's protocol (Amersham Pharmacia Biotech). The purified (RGD)_3_-tTF was analyzed by SDS-PAGE. The presence of tTF moiety in fusion protein was further confirmed by Western blotting analysis. Briefly, the proteins in the SDS-PAGE gel were transferred to a nitrocellulose membrane (Micron Separations, Inc.) and incubated sequentially with anti-human TF antibody (Sigma-Aldrich) and RGD antibody (Abcam), biotinylated secondary antibody, HRP-conjugated streptavidin, and 4-chloro-1-naphthol to identify those bands containing the tTF moiety. 

### 2.5. Labeling Fusion Protein with RBITC

According to the manufacture's protocol, the purified (RGD)_3_-tTF, tripeptide Arg-Gly-Asp (RGD) (Sigma-Aldrich, Saint Louis, MO, USA), and tissue factor (Prospect, East Brunswick, NJ, USA) were dialyzed against 0.5 M carbonate buffer (pH 9.0) and incubated with rhodamine isothiocyanate B (RBITC, Biochemika) at a molar ratio of 1 : 24 for 90 min at room temperature with end-to-end mixing. After incubation, the free RBITC was removed from the labeled (RGD)_3_-tTF, RGD, and TF by extensive dialysis against PBS pH 7.4. All the above treatments were performed under light-protected conditions. 

### 2.6. Clotting Test

Referring to coagulation experiments of Haubitz and Brunkhorst [21], fresh mouse blood was treated with 3.8% sodium citrate. Then, the blood sample was centrifuged at 4000 r/min, and the plasma was collected and used for further test. Plasma sample was added to wells of 96-well microplate (30 *μ*L/well). (RGD)_3_-tTF, TF, or RGD in a series of concentrations of 0, 0.75, 1.5, 3, and 6 *μ*mol/L was added to the wells (50 *μ*L/well). Calcium chloride (CaCl_2_) in concentration of 12.5 mmol/L was then added to the wells (20 *μ*L/well). The time from the moment of adding CaCl_2_ to the plasma to the moment of plasma clotting was recorded at room temperature. The samples without CaCl_2_ were used as controls.

### 2.7. Factor X (FX) Activation

The complex of TF and F VII can activate FX to decompose S2222 (a complex of peptide nitroaniline) into peptide and p-nitroaniline. p-Nitroaniline has an absorption peak at 405 nm. So, detecting the OD value of p-nitroaniline at 405 nm can indirectly indicate the activity of TF activating FX. Briefly, (RGD)_3_-tTF, TF, or RGD in three aminomethane (Tris) buffer solutions in a series concentration of 0.01, 0.1, 1, and 10 *μ*mol/L containing 100 nmol/L F VII were added to 96-well microplate (50 *μ*L/well), respectively, and kept at 37°C for 10 min. (RGD)_3_-tTF, RGD, or TF without F VII in concentration of 0.01, 0.1, 1, and 10 *μ*mol/L was used as controls. Then, 10 *μ*L factor X (Sigma-Aldrich) was add to wells at a concentration of 5 nmol/L. The 96-well microplate was kept at room temperature for 10 min. Thereafter, 10 *μ*L 100 mmol/L ethylenediaminetetraacetic acid (EDTA) was added to wells to terminate the reaction, and 10 *μ*L 2 nmol/L chromogenic substrate (S2222) (sigma) was then added. Absorbance at 405 nm was measured within three minutes using a microplate reader (Bio-Rad, Hercules, CA, USA).

### 2.8. Analysis of Specific Binding of (RGD)_3_-tTF and *α*_*v*_ *β*_3_

According to the method referring to Kessler et al. [8], the ability of (RGD)_3_-tTF fusion protein specifically binding with *α*_*v*_ *β*_3_ was analyzed by indirect enzyme-linked immunosorbent assay (ELISA). 96-well microplate which was coated with *α*_*v*_ *β*_3_ (5 *μ*g/L, 50 *μ*L/well) was kept at 4°C overnight and then blocked with 1% bovine serum albumin (BSA, 200 *μ*L/well). 50 *μ*L (RGD)_3_-tTF fusion protein in a series concentration of 0.012, 0.025, 0.05, 0.10, 0.20, and 0.40 *μ*mol/L was then added to the wells. The microplate was kept at 4°C for 12 hours. RGD or TF in concentration of 0.012, 0.025, 0.05, 0.10, 0.20, and 0.40 *μ*mol/L was also added to the *α*_*v*_ *β*_3_-coated wells as controls. Then, biotinylated secondary antibody and HRP-conjugated streptavidin was added to wells. After 10 min coloration, reaction was terminated with tetramethylbenzidine (TMB). Absorbance at 405 nm was measured with a microplate reader (Bio-Rad, Hercules, CA, USA).

### 2.9. Animal Experiments 

#### 2.9.1. Colorectal Cancer Mice Model

All mice used in this research were cared and treated in agreement with the regulations of ethical committee of Xiamen University. BALB/C 4-week-old male nude mice, 25 ± 4 g, were purchased from the Experimental Animal Center of Xiamen University. Murine colorectal cancer cell line CT26 was cultured with RPMI1640 supplemented with 10% fetal calf serum. CT26 cells in logarithmic growth phase were trypsinized, centrifuged, and suspended in PBS. 2 × 10^6^ CT26 cells in 0.2 mL PBS were then subcutaneously transplanted in random flank of mice to measure the size of skin tumors in 2 perpendicular directions and to calculate tumor volume according to the formula (tumor volume = length × width^2^  × 0.52). As the tumor reaches 125 mm^3^, the tumor-bearing mice were used for further experiments. 

#### 2.9.2. Observation of Drug Distribution in Tumor Animal Models In Vivo

Tumor-bearing mice were divided into (RGD)_3_-tTF, RGD, TF, and saline groups (*n* = 5). The mice in each group were injected with 200 *μ*L, 50 *μ*g RBITC-labeled (RGD)_3_-tTF, RGD, TF, or 200 *μ*L saline through tail veins. The mice without tumor as sham group were injected with 50 *μ*g RBITC-labeled (RGD)_3_-tTF through tail vein. One hour after drugs administration, the RBITC-labeled drugs were traced in the living mice using fluorescent imager (KODAK Image Station 2000MM). Twenty-four hours after being treated with (RGD)_3_-tTF, RGD, TF, or saline, the mice were sacrificed. Samples of liver, lung, kidney, brain, and tumor tissue were collected, frozen in liquid nitrogen, cut into 5 *μ*m sections, stained with Hoechst 33258, and then observed using a laser confocal microscope (FV1000, OLYMPUS) to trace the fluorescent drugs. 

#### 2.9.3. Tumor-Bearing Mice Model Treated with (RGD)_3_-tTF

Mice with skin tumor were divided into four experimental groups: (RGD)_3_-tTF, RGD, TF, and saline (*n* = 5). 50 *μ*g in 200 *μ*L saline (RGD)_3_-tTF, RGD, TF, or 200 *μ*L saline was injected into the tumor-bearing mice through the tail veins. The mice were routinely checked, the tumor's length and width were determined, and the tumor's volumes were then calculated. Ten days after drugs administration, the mice were sacrificed, and the samples of tumor tissue, heart, liver, spleen, lung, kidney, and brain tissues were collected, fixed in 10% neutral formalin, and embedded in paraffin. The samples were cut into 4 *μ*m sections for hematoxylin-eosin (HE) staining analysis to detect the tumor tissue necrosis and thrombosis in vessels with light microscope (Nikon Eclipse 50i).

#### 2.9.4. Survival Time of (RGD)_3_-tTF-Treated Tumor-Bearing Mice

Mice with skin tumor were divided into four experimental groups: (RGD)_3_-tTF, RGD, TF, and saline (*n* = 15). 50 *μ*g in 200 *μ*L saline (RGD)_3_-tTF, RGD, TF, or saline was injected through the tail veins of skin tumor-bearing mice. The survival time of mice in four groups was recorded.

### 2.10. Statistical Analysis

All data are expressed as mean values ± SEM, and *n* represents the number of animals per experimental group. Statistical comparisons between the groups were performed by rank sum test. Differences were considered significant at *P* < 0.05.

## 3. Results 

### 3.1. Identification of Target Fusion Gene of (RGD)_3_-tTF 

**Figure 1:**
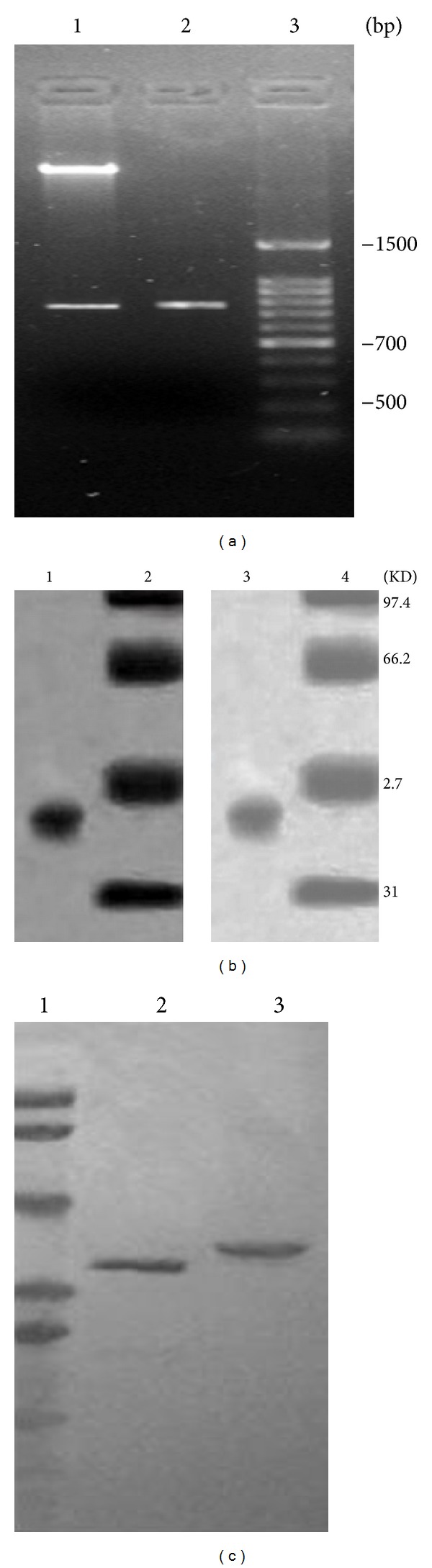
Characterization of fused gene and fusion protein of (RGD)_3_-TF. (a) PCR products of (RGD)_3_-tTF-pET22b(+); 1: PCR products of (RGD)_3_-tTF-pET22b(+) digested by restriction enzyme; 2: PCR products of gene of (RGD)_3_-tTF; 3: DNA marker. (b) Purification of (RGD)_3_-tTF. 1 and 2: SDS-PAGE; 3 and 4: Western blot; 1 and 3: (RGD)_ 3_-tTF; 2 and 4: prestained molecular weight standards. (c) Identification of purified (RGD)_3_-tTf. 1: molecular weight markers; 2: (RGD)_3_-tTF detected using the anti-TF antibody; 3: purified (RGD)_3_-tTF detected using the anti-RGD antibody.

The tTF gene in size of 657 bp was amplified and annealed with primers P_3_ containing (RGD)_3_-4C to obtain the template of fusion gene of (RGD)_3_-tTF by PCR. Then, the template of fusion gene of (RGD)_3_-tTF was added with Nco I and Xho I endonuclease sites. The expression vector pET22b(+) containing (RGD)_3_-tTF gene was reconstructed and then digested with the Nco I and Xho I restriction enzyme for further identification. The digested products of reconstructed vector were used for 1% agarose gel electrophoresis analysis. There was a single 780 bp band which was consistent with the theoretical calculated value of the gene of (RGD)_3_-tTF (784 bp) (Figure 1(a)). The clone gene sequence was identified of being consistent with target gene nucleotide sequence with ampicillin resistance selection and PCR. 

### 3.2. Expression, Purification, and Identification of (RGD)_3_-tTF

The fusion protein of (RGD)_3_-tTF was expressed by *E. coli *BL21 (DE_3_). After being purified by nickel column and SDS-PAGE electrophoresis, a single band at the size of approximately 38 kDa was found. The protein was confirmed of containing tTF and RGD motif with Western blot (Figures 1(b) and 1(c)).

### 3.3. Clotting Test

**Figure 2:**
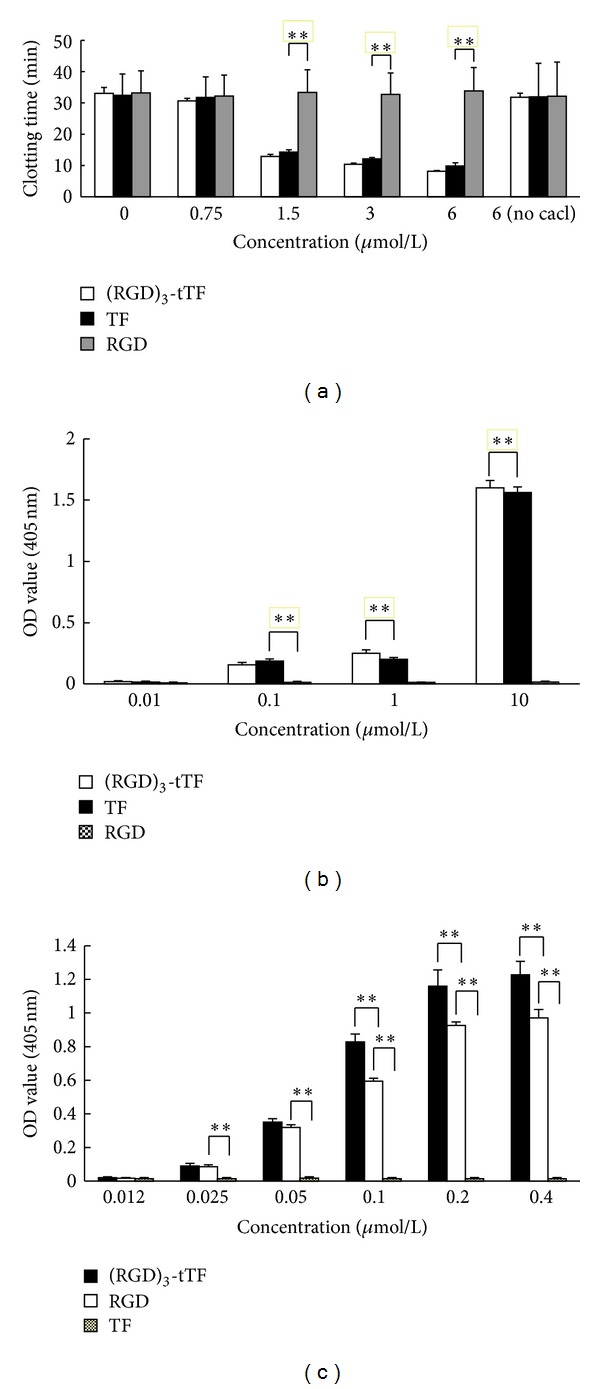
Bioactivity of (RGD)_3_-tTF. (a) Clotting time. The clotting time of (RGD)_3_-tTF was similar to that of TF but significantly higher than that of RGD; there was no significant difference between (RGD)_3_-tTF and TF (*P < 0.05,  **P < 0.01). (b) Factor X (FX) activation. At 1 *μ*mol/L or higher concentration, the activation FX by (RGD)_3_-tTF is comparable to that of free TF but significantly higher than that of RGD (*P < 0.05, **P < 0.01). (c) Specific binding to *α*_*v*_ *β*_3_. (RGD)_3_-tTF binding with *α*_*v*_ *β*_3_ was significantly higher than that of RGD and TF (*P* < 0.01), and RGD binding with *α*_*v*_ *β*_3_ was significantly stronger than that of TF (*P < 0.05,  **P < 0.01).

Plasma with sodium citrate, solely supplemented with CaCl_2_, (RGD)_3_-tTF, RGD, or TF, did not coagulate within 30 min. With the addition of CaCl_2_, the fusion protein of (RGD)_3_-tTF could effectively enhance plasma coagulation. With the concentration of (RGD)_3_-tTF increasing, the clotting time reduced accordingly. The clotting time of TF was similar to that of (RGD)_3_-tTF (*P* > 0.05) but significantly less than that of RGD (*P* < 0.05) (Figure 2(a)). 

### 3.4. F X Activation

A series of concentrations of (RGD)_3_-tTF, TF, and RGD were used for activation analysis. Absorbance at 405 nm was measured after activating FX. (RGD)_3_-tTF at 1 *μ*mol/L or higher concentration could activate FX. The FX activation ability of (RGD)_3_-tTF was comparable with that of TF (*P* > 0.05), while the activation ability of RGD in corresponding concentration was much less than that of TF and (RGD)_3_-tTF (*P* < 0.05) (Figure 2(b)).

### 3.5. Specific Binding with *α*_*v*_ *β*_3_

The specific binding of (RGD)_3_-tTF and *α*_*v*_ *β*_3_ was detected by ELISA. Both (RGD)_3_-tTF and RGD could specifically bind with *α*_*v*_ *β*_3_. The binding was dose-dependent and presented saturated phenomenon with drugs dose increasing. At the same molar concentration, the binding with *α*_*v*_ *β*_3_ of (RGD)_3_-tTF was significantly higher than that of RGD and TF (*P* < 0.01), and the binding with *α*_*v*_ *β*_3_ of RGD was significantly stronger than that of TF (*P* < 0.01). At 0.2 *μ*mol/L concentration, (RGD)_3_-tTF presented the highest binding activity with *α*_*v*_ *β*_3_, which was comparable to that of (RGD)_3_-tTF in the concentration of 0.4 *μ*mol/L (*P* > 0.05)  (Figure 2(c)).

### 3.6. Tracing of (RGD)_3_-tTF In Vivo

**Figure 3:**
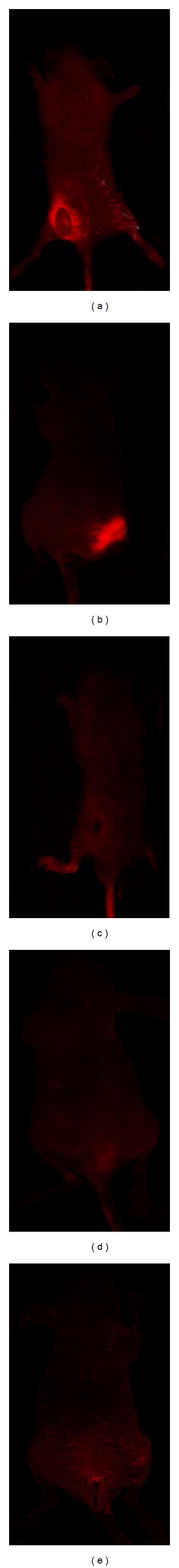
Tracing of fluorescently labeled drugs in vivo. (a) (RGD)_3_-tTF; (b) RGD; (c) TF; (d) normal mice injected with (RGD)_3_-tTF; (e) saline.

One hour after intravenously injecting (RGD)_3_-tTF or RGD, an obviously fluorescence enrichment was observed in the location of skin tumor in tumor-bearing mice (Figures 3(a) and 3(b)), while the fluorescence enrichment was not found in the other parts of the mice. No fluorescence enrichment was found in the mice injected with TF or saline (Figures 3(c) and 3(d)). No fluorescence enrichment was observed in normal mice injected with (RGD)_3_-tTF (Figure 3(e)). 

### 3.7. Tracing of (RGD)_3_-tTF in Other Tissues or Organs

**Figure 4:**
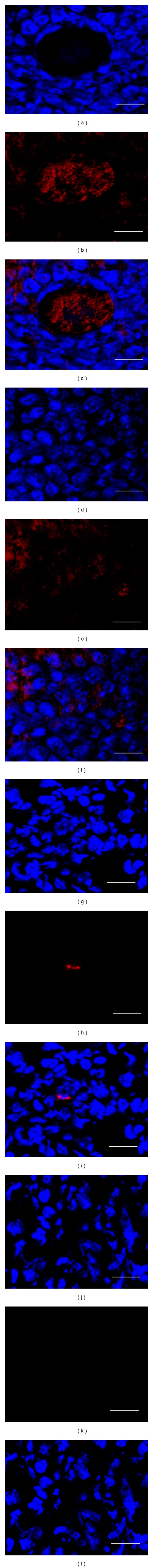
Fluorescence drugs accumulation in tumor tissue. In (RGD)_3_-tTF mice, (a) Hoechst 33258-stained nucleus (blue); (b) (RGD)_3_-tTF accumulated (red); merger of (a) and (b). In RGD mice, (d) Hoechst 33258-stained nucleus (blue); (e) RGD accumulated; (f) merger of (d) and (e). In TF mice, (g) Hoechst 33258-stained nucleus (blue); (h) no TF accumulation; (i) merger of (g) and (h) In saline mice, (j) Hoechst 33258-stained nucleus (blue); (k) no fluorescence; (l) merger of (j) and (k) (bar = 20 *μ*m).

**Figure 5:**
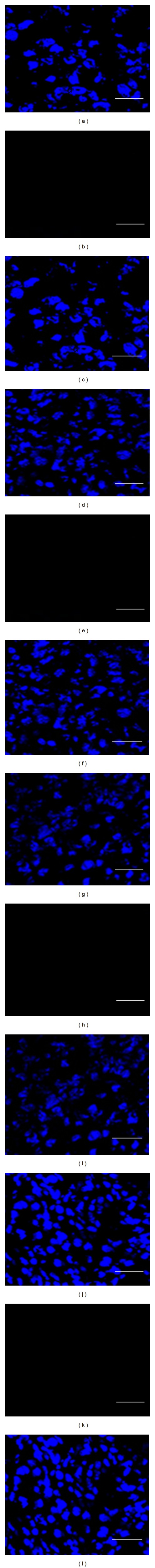
(RGD)_3_-tTF tracing in other tissues. Hoechst 33258-stained nucleus of samples of brain (a), liver (d), kidney (g), and lung (j). No fluorescence was found in samples of brain (b), liver (e), kidney (h), and lung (k). Mergers of (a) and (b) (c), (d) and (e) (f), (g) and (h) (i), and (j) and (k) (l) (bar = 20 *μ*m).

Twenty four, hours after injecting (RGD)_3_-tTF, TF, or RGD through tail vein, the samples such as tumor, liver, lung, brain, and kidney were made into sections and observed with confocal microscope. RBITC labeled (RGD)_3_-tTF or RGD was found within tumor blood vessels or in the surrounding tissue of tumor blood vessels (Figures 4(a)–4(f)). Sample sections of mice treated with free TF were not detected of the fluorescence (Figures 4(g)–4(l)). No fluorescence was found in tissues such as liver, lung, brain, and kidney of tumor-bearing mice (Figure 5). 

### 3.8. Histological Analysis

**Figure 6:**
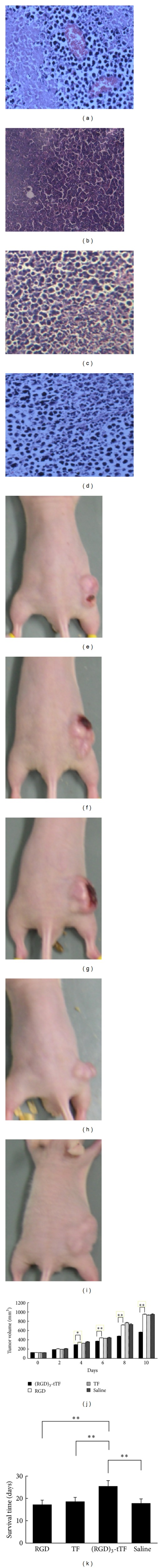
Antitumor effect in vivo. Upper panel: histological analysis of tumor tissues in mice treated with drugs. (a) Necrosis and thrombosis were found in tissues of mice treated with (RGD)_3_-tTF; (b) no necrosis and thrombosis in mice treated with TF; (c) no necrosis and thrombosis in mice treated with RGD; (d) no necrosis and thrombosis in mice treated with saline (bar = 50 *μ*m). Middle panel: tumor necrosis after treatment. (e) Necrosis was found at 4 days after injection of (RGD)_3_-tTF; (f) necrosis was found at 6 days after injection of (RGD)_3_-tTF; (g) necrosis was found at 8 days after injection of (RGD)_3_-tTF; (h) no necrosis in mice treated with TF; (i) no necrosis in mice treated with RGD. Lower panel: (j) tumor volume of mice treated with drugs. The tumor volume in mice treated with (RGD)_3_-tTF was significantly smaller than that in mice treated with TF, RGD, or saline; there was no significant difference between tTF and RGD (*P < 0.05, **P < 0.01). (k) Surviving time of tumor-bearing mice. Surviving time of tumor-bearing mice treated with (RGD)_3_-tTF was significantly longer than that in TF or RGD groups; there was no significant difference between tTF and RGD (*P < 0.05, **P < 0.01).

Thrombosis was found within the tumor blood vessels of tumor-bearing mice which were injected with (RGD)_3_-tTF (Figure 6(a)). The tumor necrosis occurrences were observed within tumor tissues. No thrombosis and necrosis were found in tumor tissue of mice which were injected with RGD or TF (Figures 6(b) and 6(c)). 

### 3.9. Therapeutic Efficacy of (RGD)_3_-tTF

After administration of drugs, the tumor of mice treated with (RGD)_3_-tTF occurred necrosis at 4 days (Figures 6(e)–6(i)). The tumor growth in mice treated with (RGD)_3_-tTF was significantly slower than that in mice treated with TF, RGD, or saline (*P* < 0.05). The tumor volume in (RGD)_3_-tTF group was significantly smaller than that of TF, RGD, or saline group (*P* < 0.05). After the absence of targeting function, TF could not inhibit tumor growth. The growth and volume of tumors in mice treated with TF were similar to that of RGD mice (*P* > 0.05) (Figure 6(j)). 

### 3.10. Survival Time of Tumor Mice Models

The survival time of tumor-bearing mice treated with (RGD)_ 3_-tTF was 25.5 ± 2.5 days and significantly longer than that of mice treated with TF (18.6 ± 1.9 days) or RGD (17.3 ± 1.9 days) (*P* < 0.05). There was no significant difference of surviving time between TF and RGD groups (Figure 6(k)).

## 4. Discussion 

In this study, we generated a fusion gene of (RGD)_ 3_-tTF which was composed of two components. Firstly, the (RGD)_ 3_ was for targeting integrin *α*_*v*_ *β*_3_ receptor of tumor blood vessels; secondly, the tTF was for thrombogenic activation. (RGD)_3_-tTF fusion protein was expressed in *Escherichia coli* strain and purified through nickel column and SDS-PAGE electrophoresis. The fusion protein of (RGD)_3_-tTF was proved of being effective in inducing blood coagulation, activating FX, and specifically binding with *α*_*v*_ *β*_3_ receptor in vitro analysis. (RGD)_3_-tTF also presented an effective role of antitumor therapy by inducing thrombosis within tumor blood vessels, blocking tumor blood supply, and causing tumor necrosis to reduce the size and volume of colorectal cancer and improve the survival time of tumor-bearing mice. 

Normal coagulation activation proceeds through the tissue factor- (TF-) dependent pathway, whereby TF forms a 1 : 1 stoichiometric complex with native factor (F) VII (F VII–TF) [22]. F VII binds to the lipid portion of TF in the presence of calcium, which acts as a bridge between TF and F VII. The TF–F VII complex activates FX and FIX to cause blood coagulation [22]. In 1997, Huang et al. [11] explored Ab-tTF complex, a new strategy of molecular targeting therapy aiming of tumor vascular network [11]. The complex of Ab-tTF, in which tTF acted as the effect factors for inducing tumor blood vessel thrombosis and MHCII antibody (Ab) acted as the tTF carrier, was prepared for targeting tumor vasculature, inducing tumor vascular thrombosis, and leading to tumor necrosis [11, 12]. Although Ab-tTF fusion protein can induce tumor vascular thrombosis and inhibit tumor growth to various degrees, the relatively large antibody molecules of Ab-tTF likely cause steric hindrance, affect the binding with markers on endothelium, and reduce the efficiency of inducing thrombosis of tumor blood vessels [23]. 

RGD-4C is a kind of small molecular polypeptide and has affinity to *α*_*v*_ *β*_3_ receptor [24]. The size of RGD-4C is smaller than that of antibody. RGD-4C could overcome the steric hindrance caused by large antibody molecules. RGD polypeptide could specifically recognize and bind to *α*_*v*_ *β*_3_ receptor [25]. Integrin *α*_*v*_ *β*_3_ receptor is rare in the blood vessels of normal tissues, and its state increases on the membrane of tumor vascular endothelial cells [26]. RGD polypeptide was used as the drug carrier by targeting the integrin *α*_*v*_ *β*_3_ receptor for therapy of tumor [8, 27, 28]; however, a previous study in which RGD-4C with a single ligand was used as the specific carrier of effect factor tTF showed that RGD-4C only induced thrombosis in small vessels [29] and suggested that the antitumor effect of RGD-4C was not ideal. The reason may be due to the fact that the affinity between the single RGD ligand and *α*_*v*_ *β*_3_ receptor was lower than that between antigen and antibody.

Studies have shown that multiple RGD-4C peptide sequences have thier unique advantages and the multiple RGD-4C with double loop and two disulfide bonds have 20% to 40% higher affinity on *α*_*v*_ *β*_3_ receptor than the single disulfide RGD does and have 200% higher affinity on *α*_*v*_ *β*_3_receptor than the linear RGD does [30]. However, it was found that the binding between the fusion protein of (RGD)_2_-tTF (double RGD peptide) and *α*_*v*_ *β*_3_ receptor for targeting the tumor vasculature was not significantly stronger than the binding of RGD-tTF and *α*_*v*_ *β*_3_ receptors [31]. Computer-assisted analysis revealed that when the number of RGD peptides was more than three, the conformation of functional domains will block each other and interfere with RGD binding with *α*_*v*_ *β*_3_ receptor [32, 33]. Therefore, in this research, we introduced the gene of three RGD-4C sequences by the 5′ end of tTF gene to produce (RGD)_3_-tTF fusion gene and (RGD)_3_-tTF fusion protein. (RGD)_3_-tTF fusion protein with two disulfide bonds and three RGD ligands enhanced the affinity on the *α*_*v*_ *β*_3_ receptor without causing excessive steric hindrance and selectively increased the efficiency of inducing tumor blood vessels thrombosis [27, 34]. In in vitro coagulation experiments, the blood coagulation capacity of (RGD)_3_-tTF fusion protein was similar to that of TF. The blood clotting time shortened with a corresponding increase of the concentration of (RGD)_3_-tTF. The results suggested that three RGD did not affect the blood coagulation of TF. There was no significant difference between (RGD)_3_-tTF and TF on blood clotting function. (RGD)_3_-tTF was capable of automatically assembling in the tumor blood vessels, while TF without RGD-4C carrier could not target tumor tissue. These results indicated that (RGD)_3_-tTF fusion protein could specifically gather in colorectal tumor blood vessels through the specific binding of RGD peptides and *α*_*v*_ *β*_3_ receptors of tumor vessels, while the free TF without RGD peptides guiding delivery could not assemble at the tumor sites.

Histological analysis showed that the tumor vascular thrombosis and part of the tumor necrosis had occurred within the tumor of mice treated with (RGD)_3_-tTF fusion protein. There were no thrombosis and necrosis in the tumor of mice treated with RGD and TF. The size and volume of tumor of mice treated with (RGD)_3_-tTF fusion protein were significantly smaller than that in mice treated with TF and RGD at all time points. Both (RGD)_3_-tTF and RGD could gather in tumor tissues; however, only (RGD)_3_-tTF induced the tumor vascular thrombosis and tumor necrosis. The occurrence of tumor vascular thrombosis and tumor necrosis was not found in normal tissue. There was scarce thrombosis that occurred in the tumor of mice treated with TF. By specific binding with *α*_*v*_ *β*_3_ receptor, the fusion protein of (RGD)_3_-tTF targetingly delivered TF to the tumor vasculature of colorectal cancer, selectively induced thrombosis of tumor blood vessels, caused necrosis of tumor tissue, and inhibited tumor growth without causing blood coagulation and necrosis in normal tissues and organs. 

In conclusion, we have shown that (RGD)_3_-tTF, which targets integrin *α*_*v*_ *β*_3_ receptor of vasculature endothelium in colorectal tumor, could induce extensive thrombosis in tumor blood vessels and lead to effective antitumor therapy in colorectal cancer-bearing mice models. Although the strategy that (RGD)_3_ peptide-mediated tTF induces tumor vasculature thrombosis and tumor necrosis by blocking blood vessel is still at the experimental stage, it is a promising treatment for future colorectal cancertherapy.
